# Whole‐Genome Sequence Profiling of *Listeria innocua From* Different Sources: Implications for Public Health

**DOI:** 10.1002/mbo3.70274

**Published:** 2026-03-20

**Authors:** Christ‐Donald Kaptchouang Tchatchouang, Daniel Jesuwenu Ajose, Giulia Amagliani, Collins Njie Ateba

**Affiliations:** ^1^ Food Security and Safety Focus Area, Faculty of Natural and Agricultural Sciences North‐West University Mmabatho South Africa; ^2^ School of Biology and Environmental Sciences, Faculty of Agriculture and Natural Sciences, Antimicrobial Resistance and Phage Biocontrol Research Group (AREPHABREG) University of Mpumalanga Mbombela South Africa; ^3^ Department of Biomolecular Sciences University of Urbino Carlo Bo Urbino Italy

**Keywords:** antibiotic resistance, food safety, foodborne bacterial pathogen, resistome, virulome

## Abstract

Foodborne disease outbreaks, particularly those associated with antimicrobial‐resistant (AMR) bacterial pathogens, have become an issue of severe public health concern owing to increased globalisation and active food trade among countries. These disease outbreaks include listeriosis, which can cause notable complications such as diarrhoea, headaches, and vomiting. The data generated during the South African foodborne outbreak caused by AMR *Listeria monocytogenes* led to the inclusion of listeriosis on the South African list of mandatory notifiable medical conditions. Prospective solution to managing the increasing threat caused by AMR foodborne pathogens to humans require frequent surveillance of food products using techniques with high throughput and discriminatory potential. Thus, this study assessed the virulome, resistome, and phylogenetics of two *Listeria innocua* strains (LIN_NWU_CNKT and LIN5_NWU_CNKT) previously isolated from food and water samples collected in the North‐West Province, South Africa, using whole‐genome sequencing (WGS). Based on WGS analysis, the isolates were confirmed as *L. innocua*, with genomes that are closely related to previously isolated human pathogens. The genomes of these two isolates harboured virulence genes, including those responsible for adherence (*fbpA*, *inlJ*), invasion (*aut*, *inlA*), and immune modulation (*inlC*, *lntA*). In addition, the genes encoding antibiotic resistance were found in the genomes. These genes confer resistance to antibiotics such as phosphonic acid (*fosX*), lincosamide (*lin*), tetracycline (*tetM*), and glycopeptide (*vanT*). These findings highlight a crucial need to enforce standard operating procedures in food processing to reduce the spread of AMR and foodborne outbreaks.

## Graphical abstract

The studied strains, LIN_NWU_CNKT and LIN5_NWU_CNKT, exhibit invasiveness, pathogenicity, and MDR profiles. The isolation of these strains from food products and water underscores their significant public health implications, particularly in the context of the “One Health” concept. However, the findings indicate only genetic potential rather than demonstrated pathogenic behaviour.

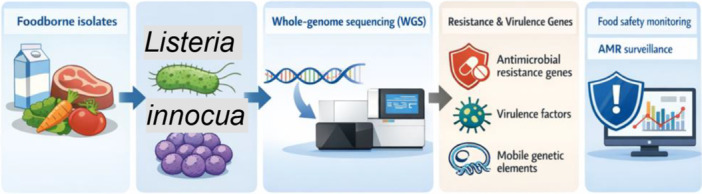

## 1 Introduction

The constant increase in the human population continues to pose significant challenges to access to safe, nutritious, and healthy food. The United Nations Sustainable Development Goals (UN SDGs), particularly Goal 3: Good Health and Well‐being for All, emphasize that food safety is a critical element for improving global health and ensuring sustainable development (Founou et al. 2021). Moreover, the desire to increase production to meet demand strains natural resources and introduces pollution to the environment, thus affecting food safety and food security (Andrade et al. 2018). Against this background, achieving an efficient and resilient food system is essential for effective food security management (The United Nations Food and Agriculture Organization FAO 2017; Garcia et al. 2020). Also, the need to urgently identify and track foodborne pathogens in the food chain, especially during outbreaks, is crucial. Traditional bacterial identification and typing methods, including serotyping, antibiotic and phage typing, are primarily based on biochemical characteristics, which can affect their sensitivity and specificity (Sloan et al. 2017). Additionally, these processes are both time‐consuming and tedious. To address these challenges, recent years have seen the development and use of more reliable molecular‐based tools that provide timely, accurate and highly effective data for the identification and tracking of bacterial contaminants in the food chain (Zhao et al. 2014; Zhao et al. 2016). These typing tools also provide data that are very useful and epidemiologically important.

Amongst the molecular typing methods, whole genome sequencing (WGS) has revolutionized the field of genomics, enabling researchers to unravel the intricate genetic makeup of microorganisms and gain invaluable insights into their biology and evolution (Fuentes‐Pardo and Ruzzante 2017). WGS offers a comprehensive analysis of an organism's entire genetic content, unveiling the complete sequence of its genome, including genes, regulatory elements, and other structural features (Fuentes‐Pardo and Ruzzante 2017; Li et al. 2021). The comprehensive genomic data obtained through WGS can be utilized to establish robust surveillance systems for monitoring the presence and spread of *Listeria* species within the food production chain (El Zowalaty et al. 2019; Gwida et al. 2020). This information empowers regulatory bodies and food manufacturers to implement targeted interventions that prevent outbreaks and safeguard public health.

*Listeria* species, particularly *L*. *innocua*, possess multiple stress response mechanisms that enable them to overcome varying environmental conditions such as temperature, osmotic pressures, and pH; thus, they can easily survive within different stages of the food chain (Saldivar et al. 2018; Wiktorczyk‐Kapischke et al. 2021; Osek et al. 2022). *L*. *innocua* is presumed to be non‐pathogenic. Nonetheless, incidences of animal listeriosis caused by *L*. *innocua*, a close genetic relative of *L*. *monocytogenes*, have been reported (Liu 2013; Matto et al. 2022). Moreover, the potential of *Listeria* species to cause severe and challenging pathological conditions, including listeriosis in the elderly, immunocompromised individuals, and pregnant women (Özgenç and Meltem 2016; Schlech 2019), further underscores its status as a notorious foodborne pathogen. Genes from different bacterial genera may have been acquired by *L*. *innocua* through lateral gene transfer, gene duplication, and gene divergence, thereby contributing to its pathogenic potential and evolution (Chiara et al. 2015).

Although *Listeria* species are not on the approved World Health Organization (WHO) global priority pathogens list, their clinical significance in South Africa is enhanced by the recent occurrence of the world's most severe listeriosis outbreak (Thomas et al. 2020). Therefore, investigating the genetic foundations of virulence and antimicrobial resistance (AMR) among *L. innocua* strains is crucial to safeguarding public health (Shoai‐Tehrani et al. 2019).

Hence, this study assessed the virulome, resistome, and phylogenetics of *L. innocua* strains previously isolated from various sources in the North‐West Province of South Africa using whole‐genome sequencing (WGS).

## 2 Materials and Methods

### 2.1 Bacterial Strains

Two *Listeria* strains (LIN_NWU_CNKT and LIN5_NWU_CNKT) previously isolated from food (meat) and water samples (Kaptchouang Tchatchouang et al. 2022), were subjected to WGS analysis.

### 2.2 Genomic DNA Extraction

Pure frozen cultures of multidrug‐resistant (MDR) *Listeria* species were revived by subculturing on a *Listeria* isolation medium (Oxford formulation, Acumedia). The plates were incubated aerobically at 37°C for 24–48 h. Pure greenish‐grey colonies were inoculated in 15 mL tryptic soy broth (TSB) (Merck, South Africa) and incubated aerobically at 37°C for 24 h. Bacterial cells were harvested through centrifugation at 10000 rpm for 10 min and the supernatant was discarded. Genomic DNA was extracted from the cells using the Research Genomic DNA^TM^–Tissue MiniPrep Kit (Zymo Research, ZR Corp. Irvine, United States). The DNA was quantified using a NanoDrop^TM^ Lite spectrophotometer (Thermo Fischer Scientific, Walton, MA, United States). DNA samples with A260/A280 ratios of 1.80–2.0 were classified as high‐purity DNA and used for sequencing.

### 2.3 Sequencing and Library Preparation

The strains' genomes were sequenced using an Illumina MiSeq platform (Illumina, United States). 1 ng of the genomic DNA was tagmented with the Nextera XT DNA library prep kit (Illumina, United States) according to the manufacturer's protocol. The kit reagents fragment the DNA while simultaneously adding adapter sequences. The libraries were amplified using a limited‐cycle PCR program (12 cycles) to add the index 1 (i7) and index 2 (i5) adapters, which contain sequences required for cluster generation on the Illumina flow cell. The library was purified using 0.6x Agencourt AMPure XP beads (Beckman Coulter, United States). The quality and sizes of the resulting DNA fragments were evaluated on a 1.5% agarose gel. The libraries were quantified with a fluorometric method (Qubit, Life Technologies, United States) and normalized to 4 nM using a standard dilution method. The libraries were pooled, denatured with 0.1 N NaOH and diluted to the final loading concentration of 12 pmol. An identically treated PhiX control was added to a final concentration of 1%. Paired‐end sequencing was performed on an Illumina MiSeq platform using the MiSeq Reagent Kit V3 (600 cycles).

### 2.4 Trimming and Assembly

The FastQC version 0.11.5 (http://www.bioinformatics.babraham.ac.uk/projects/fastqc/) was used to verify the sequence quality of the raw reads. Poor‐quality sequences and adapters were removed using Trimmomatic (v0.36) (Bolger et al. 2014). SPAdes 3.13.0 was used to do a *de novo* assembly of the trimmed (high‐quality) reads (Prjibelski et al. 2020).

### 2.5 Genome Annotation and Comparative Analysis

The draft assembled genomes of the two *Listeria* sp. were annotated using Prokka (v1.12) (Seemann 2014) on the Kbase platform (https://kbase.us/) and checked with rapid annotation using the subsystem technology (RAST) server (version 2.0) for comparison (Aziz et al. 2008; Brettin et al. 2015). The circular genome maps of the isolates were generated by uploading the annotated genome to the circular genome visualization online database (http://wishart.biology.ualberta.ca/cgview/) (Stothard and Wishart 2005). Reference genomes from National Center for Biotechnology Information (NCBI) were used through Pathosystems Resource Integration Center (PATRIC) algorithms to construct a phylogenetic tree. The reference and representative genomes most comparable to the target *Listeria* strains in this study were identified using Mash/MinHash (Ondov et al. 2016). PGfams were selected from these genomes to determine how the target genomes of interest fit into the phylogeny. Thereafter, the protein sequences from these families were aligned with the Multiple Sequence Comparison by Log‐Expectation (MUSCLE 3.8.31 “default version”) software. The nucleotides for each of these sequences were mapped to obtain the protein alignment (Edgar 2004). The combined amino acid and nucleotide alignments were concatenated into a data matrix to provide support values for the phylogenetic tree. Then, this matrix was evaluated using RaxML (Stamatakis 2014) and rapid bootstrapping (Stamatakis et al. 2008). The annotated genome sequences were submitted to GenBank to obtain accession numbers.

### 2.6 Identification of Virulome and Resistome Harboured by the Isolates

The specific isolate contigs were searched against the PathogenFinder version 1.1 database (https://cge.food.dtu.dk/services/SerotypeFinder/) on the Genomic Epidemiology Online Platform (https://www.genomicepidemiology.org/services/) to determine the pathogenicity group to which the isolates belong (Cosentino et al. 2013). The contigs of the isolates were compared against the Virulence Factor Database (VFDB) (http://www.mgc.ac.cn/VFs/) to identify virulence genes in their genomes. Additionally, the VirulenceFinder (version 2.0) database (https://cge.food.dtu.dk/services/VirulenceFinder/) was used to identify acquired virulence genes. The ARGs carried by the isolates' genomes were determined by submitting the contigs to the online Resistance Gene Identifier (RGI) program (https://card.mcmaster.ca/analyze/rgi) (Zankari et al. 2012). This application predicts antibiotic resistome(s) from protein or nucleotide data using homology and single‐nucleotide polymorphism (SNP) models, based on reference data from the Comprehensive Antibiotic Resistance Database (CARD).

## 3 Results

### 3.1 The Genomic Deoxyribonucleic Acid (DNA) and Sequence Quality

The genomic deoxyribonucleic acid (DNA) extracted from the two isolates (LIN_NWU_CNKT and LIN5_NWU_CNKT) was of good quality with A260/280 ratios of 1.80 and 2.00, respectively.

### 3.2 Genomic Assembly Features of *Listeria innocua*

The genomes of the two isolates (LIN_NWU_CNKT and LIN5_NWU_CNKT) were assembled using SPAdes. The genome lengths for the LIN_NWU_CNKT and LIN5_NWU_CNKT were 3341013 and 3558124 bp, respectively. The NCBI GenBank platform has received the genomic sequences of the two isolates (LIN_NWU_CNKT and LIN5_NWU_CNKT). The accession number JAVCAR000000000 has been assigned to LIN5_NWU_CNKT, while the accession number for LIN_NWU_CNKT has not yet been released. Table 1 summarizes the information obtained from the assembled and annotated genomes. The genomes of the strains LIN_NWU_CNKT and LIN5_NWU_CNKT contained 3,769 and 3,722 protein‐coding sequences, 48 and 45 transfer RNA sequences, and 4 and 2 ribosomal RNA sequences, respectively. The G + C contents for the isolates were 37.35% and 36.66% for LIN_NWU_CNKT and LIN5_NWU_CNKT, respectively. Isolate LIN_NWU_CNKT had 518 contigs, compared to 92 for LIN5_NWU_CNKT.

**Table 1:** Genomic assembly report of LIN_NWU_CNKT and LIN5_NWU_CNKT isolates.

Features	LIN_NWU_CNKT	LIN5_NWU_CNKT
Genome size (bp)	3341013	3558124
DNA G + C content	37.35%	36.66%
Number of contigs	1	92
Contig N50	3341013	584255
Contig L50	1	2
CDS	3597	3,722
tRNA	48	45
rRNA	5	2
Partial CDS	34	17
Miscellaneous RNA	0	0
Chromosomes	1	0
Accession number	CP168261	JAVCAR000000000

*Note:* LIN_NWU_CNKT is indicative of a complete genome, while LIN5_NWU_CNKT is indicative of a high‐quality draft genome.

The fragmentation observed in LIN5_NWU_CNKT is likely due to unresolved repetitive elements, including rRNA operons, insertion sequences, and mobile genomic regions. These elements are known to complicate complete assembly when using only short‐read sequencing. This is supported by reduced recovery of rRNA copies and incomplete CDS annotations, both indicative of assembly disruptions associated with repetitive elements rather than poor sequencing quality.

### 3.3 Genomic Annotation of Strains LIN_NWU_CNKT and LIN5_NWU_CNKT

#### 3.3.1 Protein Features on LIN_NWU_CNKT and LIN5_NWU_CNKT

Data from the annotation revealed the presence of hypothetical proteins (886 for LIN_NWU_CNKT and 1,069 for LIN5_NWU_CNKT) and proteins with assigned functions (2,883 for LIN_NWU_CNKT and 2,653 for LIN5_NWU_CNKT). The proteins with functional assignments included those with Kyoto Encyclopedia of Genes and Genomes (KEGG) pathway maps, Gene Ontology (GO) assignments, and Enzyme Commission (EC) numbers. Two categories of protein families are included in the annotation with PATRIC: genus‐specific protein families (PLfams) and cross‐genus protein families (PGfams).

#### 3.3.2 Subsystem Analysis of LIN_NWU_CNKT and LIN5_NWU_CNKT Genomes

**Figure 1:**
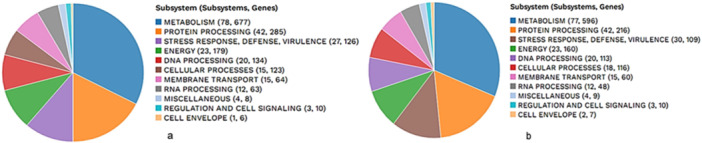
Subsystem analysis of *Listeria innocua* strain (a) LIN_NWU_CNKT and (b) LIN5_NWU_CNKT.

The subsystems unique to each of the examined genomes were outlined by PATRIC (Figure 1). The genes involved in various cellular functions were tallied up and allocated to the appropriate subsystems.

**Figure 2:**
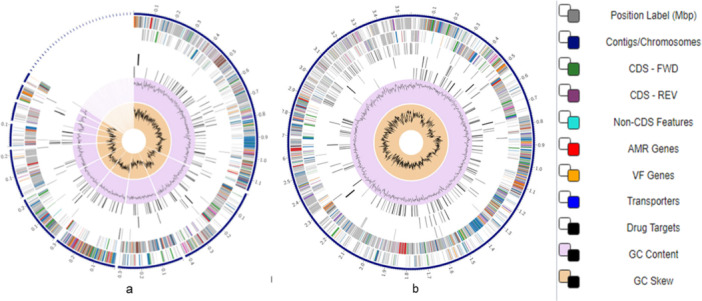
The distribution of the annotated genomes of strains LIN_NWU_CNKT (a) and LIN5_NWU_CNKT (b) in a circular graphical display. This display comprises, in order from the outer to the inner rings, contigs, CDS on the forward and reverse strands, RNA genes, CDS with homology to known antimicrobial resistance genes, CDS with homology to known virulence factors, GC content, and GC skew. The subsystem to which these genes belong is indicated by the colours of the CDS on the forward and reverse strands (see legend).

A circular graphical representation of the distribution of the bacterial genome annotations in a circular pattern is shown in Figure 2.

Functional categorization offers clues about how genome content relates to ecological adaptation. PATRIC subsystem analysis, supported by KEGG pathway mapping, showed that both isolates are enriched for genes involved in core metabolism, cell wall biogenesis, transport systems, and stress response. These categories are typical of environmental fitness and persistence rather than specialized virulence. The predicted virulence‐ and AMR‐associated genes are dispersed throughout the genome rather than organized into distinct pathogenicity islands, suggesting they form part of the conserved genomic background. GC content and GC skew profiles are relatively uniform, with no clear signatures of large, recently acquired regions, although interpretation is constrained by the fragmented assemblies. Generally, the annotations support an environmentally adapted lifestyle and suggest potential interaction‐associated traits whose functional relevance would require experimental validation.

#### 3.3.3 Virulence Genes Detected in LIN_NWU_CNKT and LIN5_NWU_CNKT Genomes

The genomes of both isolates contained multiple virulence genes, as indicated by the results from the Virulence Factors of Bacterial Pathogens online platform (Table 2). Some of the virulence genes identified in the genomes of both isolates include those involved in adhesion (*dltA*, *fbpA*, *ami*, *inlJ*, and *lap*), invasion (*hpt*, *aut*, *iap*/*cwhA*, *gtcA*, and *inlA*), peptidoglycan modification (*oatA* and *pdgA*), and surface anchoring proteins (*lgt*, *lspA*, and *srtA*). Generally, the genome of isolate LIN5_NWU_CNKT contained a higher proportion of virulence genes than that of LIN_NWU_CNKT.

**Table 2:** Virulence genes detected in LIN_NWU_CNKT and LIN5_NWU_CNKT.

Virulence factors class	Virulence factors	Related genes	
		*Listeria innocua* (LIN_NWU_CNKT)	*Listeria innocua* (LIN5_NWU_CNKT)
Adherence	d‐alanine‐polyphosphoribitol ligase	*dltA*	*dltA*
	Fibronectin‐binding protein	*fbpA*	*fbpA*
	GW autolysin	*Ami*	*ami*
	Internalin J (LPXTG protein)	*inlJ*	*inlJ*
	*Listeria* adhesion protein	*lap*	*lap*
	Ebp pili (*Enterococcus*)	*—*	*srtC1*
Bile resistance	Bile‐salt hydrolase	*—*	*bsh*
Enzyme	PI‐PLC	*plcA*	*—*
	Serine‐threonine phosphatase	*—*	*stp*
Immune modulator	InlC	*inlC*	*—*
	InlK	*—*	*inlK*
	LntA	*lntA*	*—*
Intracellular survival	Lipoate protein ligase A1	*lplA1*	*lplA1*
	Oligopeptide‐binding protein	*oppA*	*oppA*
	Post‐translocation chaperone	*—*	*prsA2*
	Sugar‐uptake system	*hpt*	*—*
Invasion	Autolysin (GW protein)	*aut*	*aut*
	Cell wall hydrolase	*iap/cwhA*	*iap/cwhA*
	Cell wall teichoic acid glycosylation protein	*gtcA*	*gtcA*
	Internalin A (LPXTG protein)	*inlA*	*inlA*
	Internalin B (GW protein)	*—*	*inlB*
	Internalin P	*inlP*	*—*
	Lipoprotein promoting entry protein	*—*	*lpeA*
	Virulence protein (LPXTG protein)	*Vip*	*—*
Iron uptake	Hemoglobin binding protein	*—*	*hbp2*
Nucleation‐promoting factor	ActA	*actA*	*—*
Peptidoglycan modification	OatA	*oatA*	*oatA*
	PdgA	*pdgA*	*pdgA*
Regulation	AgrA/AgrC	*agrA*	*agrA*
		*agrC*	*agrC*
	CheA/CheY	*cheA*	*cheA*
		*cheY*	*cheY*
	LisR/LisK	*lisK*	*lisK*
		*—*	*lisR*
	Positive regulatory factor	*prfA*	*—*
	VirR/VirS	*virR*	*virR*
		*virS*	*virS*
Surface protein anchoring	Lipoprotein diacylglyceryl transferase	*Lgt*	*lgt*
	Lipoprotein‐specific signal peptidase II	*lspA*	*lspA*
	Sortase A	*srtA*	*srtA*
	Sortase B	*—*	*srtB*

#### 3.3.4 Antibiotic Resistant (AR) Genes of Listeria innocua Strain LIN_NWU_CNKT and LIN5_NWU_CNKT

Analysis of the genomes for ARGs revealed the presence of several genes. The genomes of both strains possessed phosphonic acid, lincosamide, glycopeptide, and tetracycline resistance genes. Details of the antibiotic classes and the corresponding drugs for which resistance determinants were detected in the genomes are presented in Table 3. The mechanisms of resistance for the ARGs that were identified in these genomes are associated with either target antibiotic efflux pumps [*tet(45)* and *tet(M)*], alteration of the target antibiotic binding site (*vanT* and *vanY*) or antibiotic inactivation enzymes (*FosX, Fosl,* and *lin*).

**Table 3:** Antibiotic resistance detected in strains LIN_NWU_CNKT and LIN5_NWU_CNKT.

**Drug class**	**AMR gene**	**AMR gene family**	**Resistance function**	**LIN_NWU_CNKT**	**LIN5_NWU_CNKT**
Phosphonic acid antibiotic	*FosX*	Fosfomycin thiol transferase	Antibiotic inactivation	+	+
Lincosamide antibiotic	*Lin*	Lincosamide nucleotidyltransferase (LNU)	Antibiotic inactivation	+	+
Glycopeptide antibiotic	*vanT* gene in *vanG* cluster	Glycopeptide resistance gene cluster, vanT	Antibiotic target alteration	+	+
Glycopeptide antibiotic	*vanY* gene in *vanB* cluster	vanY, glycopeptide resistance gene cluster	Antibiotic target alteration	+	+
Tetracycline antibiotic	*tet(45)*	Major facilitator superfamily (MFS) antibiotic efflux pump	Antibiotic efflux	—	+
Tetracycline antibiotic	*tet(M)*	Major facilitator superfamily (MFS) antibiotic efflux pump	Antibiotic efflux	—	+
Phosphonic acid antibiotic	*Fosl*	Fosfomycin thiol transferase	Antibiotic inactivation	—	+

#### 3.3.5 Phylogenetic Assessment of Nucleotide Sequences of Strains LIN_NWU_CNKT and LIN5_NWU_CNKT

**Figure 3:**
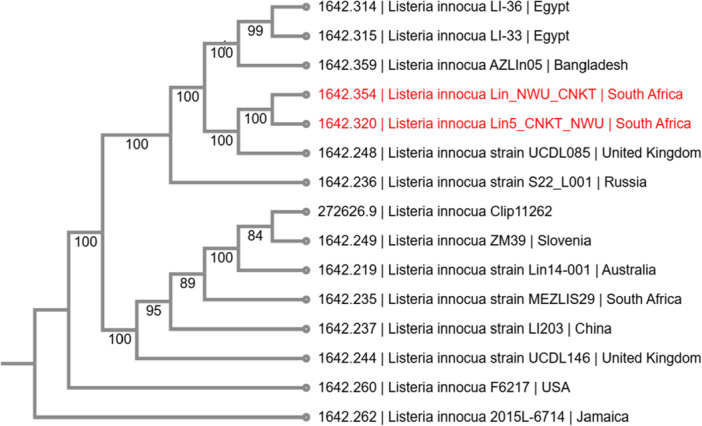
Phylogenetic tree determining the relationship between strains LIN_NWU_CNKT and LIN5_NWU_CNKT, and other *Listeria* isolates.

The phylogenetic tree, which reveals the evolutionary relationship between the genomes studied and other selected strains from similar sources across continents, is presented (Figure 3). The PATRIC method employ NCBI reference genomes to create a phylogenetic tree. Mash/MinHash was used to identify the representative and nearest reference genomes to our strains of interest.

## 4 Discussion

Food safety, which involves implementing strategies and processes to safeguard the food supply chain from contamination by microbial and chemical pollutants, is considered an important public health issue worldwide (Gizaw 2019; Lebelo et al. 2021). Even though many countries throughout the world may tend to increasingly operate interdependently with respect to their available food supply chains and food safety, the increasing occurrence of foodborne outbreaks, especially due to increased globalization, illustrates the importance of strict control systems (Scallan et al. 2011; Jacobsen 2018; Pozio 2020).

Foodborne pathogens, including but not limited to *L. monocytogenes*, *Escherichia coli*, *Staphylococcus aureus*, *Salmonella* species, *Campylobacter* spp. and *Clostridium perfringes* are considered a significant threat to food safety (Zhao et al. 2017; Gourama 2020; Vieira et al. 2022). Moreover, with the drive by the WHO to ensure “good health and well‐being for all” through the Sustainable Development Goals (Goal 3), it is reported that food safety is a critical element to assist in realizing this aim (Founou et al. 2021). Given that food supply chains operate across multiple local, national, and international borders, the involvement of decision‐makers and all stakeholders, as well as adherence to effective and efficient measures on farms, in meat processing facilities, retail supermarkets, at street vendors, and in homes, is critical (World Health Organization WHO 2015). Moreover, the utilization of highly reliable, sensitive, and specific identification assays coupled with typing methods with high recognition abilities and high repetition rates are important to ensure consistency for epidemiological purposes (van Belkum et al. 2007).

Data from bacterial pathogen virulence factor databases confirmed that the LIN_NWU_CNKT and LIN5_NWU_CNKT genomes harbour a wide range of virulence genes. The virulence factors harboured by the two isolate genomes include adherence (*fbp*A, *inlJ*, *lap*), invasion (*inlA*, *aut*, *iap*/*cwhA*), iron uptake (*hbp2*), intracellular survival (*opp*A, *hpt*, *prsA2*), and immune modulation (*inl*C, *inlK*, *lntA*). Several classes of extracellular organelles in bacteria, which facilitate attachment and motility, have been associated with virulence factors that contribute to pathogenicity (Pakbin et al. 2021). These extracellular organelles include flagella, pili, and curli fibres, which are classified as adherence virulence factors. Notably, the *prfA* gene was found in the LIN_NWU_CNKT genome in this investigation. In pathogenic *Listeria* species, the expression of virulence genes, including *prfA*, depends on the prfA protein and is essential under high‐temperature and stressful conditions (Gaballa et al. 2019). Furthermore, both research strains carried the internalin A (*inlA*) gene, which is noteworthy given its role in bacterial adhesion to intestinal cells. The present study supports the findings of Drolia and Bhunia (2019), who also identified the *inlA* gene in the samples they examined. Ferreira da Silva et al. (2017) conducted a study examining the occurrence of premature stop codons (PMSCs) in the *inl*A gene. This mutation type reduces virulence and is more common in food isolates than in clinical isolates. While antibiotics prescribed for the treatment of infections in humans may not be utilized, they nonetheless can significantly influence the emergence of resistance and virulence strains. The existence of the examined MDR strains in ready‐to‐eat foods warrants significant attention. These species may serve as reservoirs for ARGs, facilitating their dissemination to other bacteria through mobile genetic elements, with the potential co‐transfer of virulence‐associated traits (Jaber et al. 2025).

Antibiotics such as ampicillin, tetracycline, and sulfamethoxazole are commonly used to treat listeriosis (Thønnings et al. 2016). This investigation found resistance to the novel phosphonic antibiotic classes, including glycopeptides, lincosamides, and tetracyclines. One key public health concern is antibiotic resistance, particularly regarding tetracycline and the new class of phosphonic acid antibiotics. Several authors have reported resistance to erythromycin and tetracycline in *Listeria* spp. isolated from raw and cooked meats and fish products (Arslan and Baytur 2019; Maćkiw et al. 2020). However, this study reported only resistance to tetracycline, unlike the aforementioned. Nonetheless, a rise in staphylococcal strains of animal origin has been shown to exhibit a unique resistance phenotype, phenotypic lincosamide resistance/macrolide susceptibility (LR/MS), which appears to be associated with the *lnuA* and *lnuB* genes (Arana et al. 2014; Ajose et al. 2024). These genes are typically found on small plasmids, and research has confirmed that they can transpose between bacterial genera of animal and human origin (Escolar et al. 2017; Adekanmbi et al. 2024). Thus, the *lin* gene, which encodes lincosamide resistance, may have been acquired by horizontal gene transfer from other bacteria of different species. Mobile genetic elements (MGEs), such as plasmids, insertion sequences, and transposons, can facilitate the transmission of acquired genes.

AMR is on the rise and has major implications for the management of infectious diseases, as bacteria can develop or acquire resistance mechanisms through the transfer of genetic material from other bacterial species. Additionally, this investigation identified resistance genes involving antibiotic efflux (*tetM*), antibiotic target modification (*vanTYGB*), and antibiotic inactivation (*lin*, *fosX*). This observation negates the report of Wilson et al. (2018). In their study, Wilson et al. (2018) reported that all strains had the fosfomycin (*fosX*) resistance gene; however, no genes linked to erythromycin (*ermABC*) or tetracycline (*tetA*) were found. Nonetheless, our results align with those of Mafuna et al. (2021), who reported that resistance genes such as *fosX*, *lin*, *mprF*, and *norB* were identified in the examined strains.

In terms of evolutionary relationship, although the comparative strain, *Listeria innocua* strain UCDL085, a presumed non‐persistent contaminant, was originally isolated in the United Kingdom (Palaiodimou et al. 2021), phylogenomic analysis revealed close relatedness to the South African isolates (Lin_NWU_CNKT and Lin5_NWU_CNKT), indicating that *Listeria innocua* displays limited geographic structuring. This supports the idea that it is a cosmopolitan environmental species whose evolution is mostly shaped by ecological selection at soil–water interfaces rather than by being cut off from other species (Wu et al. 2019; Jespersen et al. 2023; Mo et al. 2026). The detection of closely related strains across continents emphasizes the interconnectedness of environmental microbiomes. It also underscores the necessity for integrated surveillance of non‐pathogenic *Listeria* species, which serve as reservoirs for adaptive genetic elements.

Clonal complexes retain a high degree of conservation for MGEs such as plasmids, transposons, and prophages, some of which harbour resistance genes. To survive and thrive in the food matrix or food processing environment, highly variable sites with novel genetic resistance traits may be introduced into genomes by acquiring MGEs via gene transfer (Palma et al. 2020). Hence, MGE profiling may be useful for distinguishing closely related strains and for tracking the evolutionary history of *Listeria* strains. Genetic relationships within and between species were revealed through a genome‐wide investigation. According to our findings, the strains under investigation have evolved from a non‐pathogenic strain, *L. innocua* UCDL085. The fact that the examined strains were determined to harbour pathogenic and invasive determinants validates the acquisition of these genes, a trait typical of environmental isolates. Mutations might also be responsible for them.

The lack of a gene cluster involved in the transport and metabolism of carbohydrates and amino acids distinguishes other *Listeria* species from *L. monocytogenes* (Hain et al. 2006). Unlike its pathogenic relative, *L*. *monocytogenes*, *L*. *innocua* evolves through genome plasticity without major species‐level divergence. This demonstrates a significant genomic similarity between *L. monocytogenes* and the examined genomes. Simply put, the AMR‐associated genes suggest that the detected genes likely reflect intrinsic or lineage‐associated features rather than recently acquired, horizontally transferable resistance mechanisms. These observations align with reports indicating that acquired AMR is relatively uncommon in *Listeria* spp., especially in environmental isolates (Bertsch et al. 2014; Moura et al. 2024). Consequently, while these strains harbour resistance‐associated determinants, their potential to disseminate AMR to other bacterial populations and their immediate risk to human health appear limited. Localized increases in resistance have been observed under intensified antimicrobial selection, which calls for ongoing surveillance (Shaaban et al. 2025).

## 5 Conclusions and Recommendations

This study reveals the features present in the genomes of two *Listeria innocua* strains, LIN_NWU_CNKT and LIN5_NWU_CNKT, associated with their invasiveness, pathogenicity, and MDR profiles. The isolation of these strains from food products and water underscores their significant public health implications, particularly in the context of the “One Health” concept. South Africa experienced the world's most severe outbreak of listeriosis in 2017, caused by the consumption of polony contaminated with *L. monocytogenes*. Despite limited information on prevalence, occurrence, antimicrobial susceptibility patterns, biofilm‐forming ability, and its association with foodborne outbreaks of other *Listeria* species, such as *L*. *innocua*, evidence of its genetic relatedness to *L*. *monocytogenes* suggests the need for targeted investigations. Moreover, the association of *L. innocua* with some excessively “rare” complications, such as septicemia and meningitis in humans and ruminants, amplified their public health implications. In addition to the detection of several virulence factors in the *L. innocua* genomes, the isolates were classified as human pathogens and may cause diseases in susceptible hosts if consumed in food and water.

It is important to note that genomic detection alone does not equate to functional virulence. Many of these loci are widely conserved among *Listeria* spp. and may contribute to general cell wall maintenance, environmental persistence, or host interaction rather than pathogenicity per se. Without transcriptional, proteomic, or infection‐model data, it is not possible to determine whether these genes are expressed, regulated, or capable of conferring virulence *in vivo*. The findings therefore indicate genetic potential rather than demonstrated pathogenic behaviour. Further work using expression analyses, cell culture or animal models, and comparative genomics with confirmed pathogenic strains would be required to clarify the biological relevance of these determinants in *L*. *innocua*.

## Author Contributions

Conceptualisation: Christ‐Donald Kaptchouang Tchatchouang, Giulia Amagliani, and Collins Njie Ateba. Methodology: Christ‐Donald Kaptchouang Tchatchouang, Giulia Amagliani, and Collins Njie Ateba. Validation: Christ‐Donald Kaptchouang Tchatchouang, Daniel Jesuwenu Ajose, Giulia Amagliani, and Collins Njie Ateba. Investigation: Christ‐Donald Kaptchouang Tchatchouang. Writing – original draft preparation: Christ‐Donald Kaptchouang Tchatchouang. Writing – review and editing: Daniel Jesuwenu Ajose, Giulia Amagliani, and Collins Njie Ateba. Supervision: Giulia Amagliani, and Collins Njie Ateba. All authors read and approved the manuscript for publication.

## Funding

The authors received no specific funding for this work.

## Conflicts of Interest

The authors declare no conflicts of interest.

## Data Availability

The datasets generated from this study has been deposited in the NCBI database under the accession numbers “CP168261” and “JAVCAR000000000” for Lin_NWU_CNKT and Lin5_NWU_CNKT, respectively.
